# Photocrosslinked Fish Collagen Peptide/Chitin Nanofiber Composite Hydrogels from Marine Resources: Preparation, Mechanical Properties, and an In Vitro Study

**DOI:** 10.3390/polym15030682

**Published:** 2023-01-29

**Authors:** Shinya Yano, Kei Yamaguchi, Mitsuhiro Shibata, Shinsuke Ifuku, Naozumi Teramoto

**Affiliations:** 1 Department of Applied Chemistry, Faculty of Engineering, Chiba Institute of Technology, 2-17-1 Tsudanuma, Narashino, Chiba 275-0016, Japan; 2 Department of Chemistry and Biotechnology, Graduate School of Engineering, Tottori University, 4-101 Koyama-cho Minami, Tottori, Tottori 680-8550, Japan

**Keywords:** fish collagen, peptide, chitin nanofibers, hydrogel, photocrosslinking

## Abstract

Fish collagen peptide (FCP) is a water-soluble polymer with easy accessibility, bioactivity, and reactivity due to its solubility. The gelation of FCP can be carried out by chemical crosslinking, but the mechanical strength of FCP hydrogel is very low because of its intrinsically low molecular weight. Therefore, the mechanical properties of FCP gel should be improved for its wider application as a biomaterial. In this study, we investigated the mechanical properties of M-FCP gel in the context of understanding the influence of chitin nanofibers (CHNFs) on FCP hydrogels. FCP with a number average molecular weight (*M*_n_) of ca. 5000 was reacted with glycidyl methacrylate (GMA) and used for the preparation of photocrosslinked hydrogels. Subsequently, composite hydrogels of methacrylate-modified FCP (M-FCP) and CHNF were prepared by the photoirradiation of a solution of M-FCP containing dispersed CHNF at an intensity of ~60 mW/cm^2^ for 450 s in the presence of 2-hydroxy-1-[4-(hydroxyethoxy)phenyl]-2-methyl-1-propanone (Irgacure 2959) as a photoinitiator. Compression and tensile tests of the FCP hydrogels were carried out using a universal tester. The compression and tensile strength of the hydrogel increased 10-fold and 4-fold, respectively, by the addition of 0.6% CHNF (20% M-FCP), and Young’s modulus increased 2.5-fold (20% M-FCP). The highest compression strength of the M-FCP/CHNF hydrogel was ~300 kPa. Cell proliferation tests using fibroblast cells revealed that the hydrogel with CHNF showed good cell compatibility. The cells showed good adhesion on the M-FCP gel with CHNF, and the growth of fibroblast cells after 7 days was higher on the M-FCP/CHNF gel than on the M-FCP gel without CHNF. In conclusion, we found that CHNF improved the mechanical properties as well as the fibroblast cell compatibility, indicating that M-FCP hydrogels reinforced with CHNF are useful as scaffolds and wound-dressing materials.

## 1. Introduction

The world seafood market has been and still is being steadily developed due to technical developments in aquaculture and logistics [1]. Accordingly, increasing quantities of byproducts, including trimmings, fins, frames, heads, skin, and viscera from fish and shells from shellfish, are discarded by the fisheries industry [2]. To achieve sustainable development, the maximum utilization of fisheries byproducts is very important. Large quantities of chitin and collagen can be obtained from these byproducts. Fish collagen peptide (FCP) and fish gelatin can be prepared from fish scales and fish skins as unused bioresources. These polypeptides originate from collagen, which plays an important role as a structural protein in vertebrate bodies. FCP is anticipated to be a useful biocompatible and biofunctional material, as the amino acid composition and partial sequence are the same as those of collagen. Bioactive properties of FCP, such as antioxidant [3] and antihypertensive [4] properties, have been reported. Hong et al. [5] summarized the methods used for the preparation and analysis of collagen peptides, together with a brief statement about future perspectives. Morimura et al. [6] reported an effective process for the extraction of collagen hydrolyzates (FCP) from fish bone and skin, and this FCP showed significant antiradical activity and angiotensin-I-converting enzyme inhibitory activity, with the potential for reducing blood pressure. Saito et al. [7] investigated the effect of FCP from fish skins on the lipid profile in rats. They found that rat plasma triglycerides were significantly decreased 2 h after the administration of FCP. Kobayashi et al. [8] assessed the antioxidant effect of collagen peptides using electron spin resonance.

FCP is usually prepared by the enzymatic degradation of fish collagen to solubilize it, and its chain is shorter than that of fish gelatin. The food processing industry generates several types of FCP, with typical molecular weights ranging from 500 to 5000. FCP is easily prepared, but its utility for self-standing biomaterials is somewhat highly skilled because of its low molecular weight. Although biomaterials such as biodegradable wound-dressing materials and implantable scaffolds for regenerative medicine are attractive potential applications of FCP, its hydrogels exhibit low mechanical strength and very high brittleness due to the low molecular weight of FCP. Furthermore, FCP is easily soluble in water at room temperature, unlike gelatin from domestic animals, such as pigs and cows, and does not gel, even when cool. Therefore, hydrogels prepared from FCP not only require chemical or enzymatic crosslinking but also reinforcing materials. Wang et al. [9] prepared electrospun nanofibrous membranes from FCP and chitooligosaccharides for a potential application in dressing wounds. They used polyvinyl alcohol to enhance the fiber-forming ability of the material. Zhang et al. [10] prepared composite hydrogels from carboxymethyl chitosan (CMCS), FCP, and oxidized konjac glucomannan by a crosslinking reaction in which amino groups of carboxymethyl chitosan reacted with aldehyde groups of oxidized konjac glucomannan. FCP was bound to CMCS in advance using transglutaminase. Lu et al. [11] developed gastric acid-responsive composite hydrogels of *N*-acetylcysteine-grafted chitosan, tilapia collagen peptide, and alginate containing calcium carbonate. The hydrogel was successfully formed in situ by the interaction between calcium ions and alginate in the simulated gastric fluid.

In this study, we synthesized methacrylate-modified FCP (M-FCP) and crosslinked M-FCP by ultraviolet (UV) irradiation in the presence of a photo-radical initiator to yield a self-standing hydrogel. We focused on FCP due to the benefits of its valuable bioactivities, such as its antioxidative [3,8] and antihypertensive [4,6] abilities, and its ability to promote wound healing [12], repair cartilage [13], regenerate alveolar bone [14], and differentiate adipose tissue-derived stromal cells [15]. Furthermore, its water solubility at room temperature is very important to form the desired shape in a mold, without air bubbles. Despite such advantages, however, no paper has been published regarding self-standing FCP-based hydrogels from any group other than our laboratory, because the mechanical properties of hydrogels prepared from FCP are very low due to the intrinsic low molecular weight of FCP. We succeeded in their preparation using CHNF, and we report the remarkable improvement in the mechanical properties by the addition of a very small amount of CHNF. We achieved this by using only marine resources as the objective of this study, as marine resources have advantages in religious issues and when compared to materials from mammalian sources with some potential diseases, such as bovine spongiform encephalopathy (BSE).

To improve the mechanical properties of hydrogels prepared from proteins or peptides, many trials and investigations have been conducted using approaches other than crosslinking. For example, composites with nano- or micro-filler materials have been reported, such as carbon nanotubes [16], hydroxyapatites [17,18], imogolite [17], hydrotalcite [19], poly(glycolic acid) [20], or cellulose nanomaterials [21,22], topological linking [23], double networks with bacterial cellulose [24], hydroxypropyl methylcellulose [25] or pullulan dialdehyde [26], and homogeneous networks [27]. In a review paper, Thakur et al. summarized numerous research studies into gelatin hydrogel nanocomposites [28]. These trials showed that composite hydrogels are easy to prepare with other materials and many types of hydrogels can be used. Even when limited to composites of hydrogels with organic and inorganic fibrillar materials, many reports have been published [16,18,20,21,22]. We have also reported that nanofibers such as imogolite [29] and microfibrillated cellulose [30] were found to be effective in the mechanical enhancement of peptide hydrogels. However, most fibrillar materials used in previous studies were non-degradable in the human body. Although poly(glycolic acid) [20] is biodegradable, the degradation product is an acidic molecule that can elicit an inflammatory response if present in high concentrations. Among the various types of fibers, since their preparation techniques were developed and popularized, chitin nanofibers (CHNFs) have become attractive materials for biomaterial applications. In 2004, Min et al. [31] reported an electrospun CHNF with an average diameter of 110 nm. Since then, Zhao et al. [32] used an ultrasonic extraction technique to obtain nanofibers from various natural materials, including chitin fibers. The diameter of the nanofibers ranged from 30 to 120 nm. In a previous report by one of the authors (S.I.) [33], a successful and promising method for the mechanical preparation of CHNF with a uniform width of 10 to 20 nm was developed using a special grinder. Salaberria et al. [34] used a dynamic, high-pressure homogenizer for the same purpose, while Zhong et al. [35] used the deposition method to obtain self-assembled fine CHNF from chitin solutions in 1,1,1,3,3,3-hexafluoro-2-propanol or LiCl/*N*,*N*-dimethylformamide. There have also been reports regarding chitin nanowhiskers, or nanocrystals. The preparation of chitin crystalline particles was first reported by Marchessault et al. in 1959 [36]. The extraction process involved treatment with strong acid, causing partial deacetylation. Fan et al. reported a new process for the preparation of chitin nanocrystals, by TEMPO-mediated oxidation [37]. Kadokawa et al. [38] developed another new method to prepare chitin nanowhiskers, using an allyl-modified ionic liquid. CHNF can be applied in a wide range of fields, from transparent polymer composites [39] to tissue engineering [40].

**Scheme 1:**
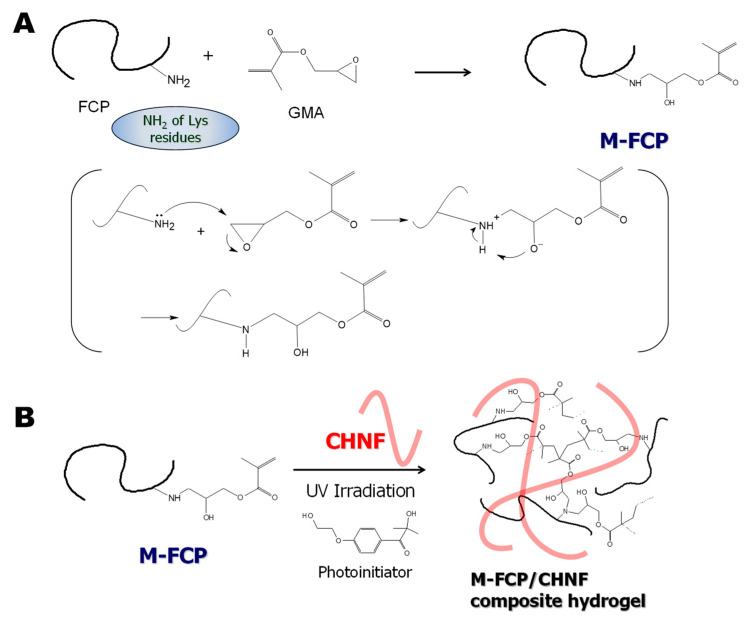
(**A**). Synthesis of M-FCP from FCP and GMA, and the reaction mechanism. (**B**). Photopolymerization of M-FCP in the presence of CHNF and a photoinitiator to obtain M-FCP/CHNF composite hydrogels.

As for the combination of CHNF and gelatin or collagen, Hassanzadeh et al. [41] prepared high-performance composite hydrogels with a higher ratio of CHNF through the self-assembly of CHNF in hexafluoro-2-propanol (HFIP) solvent. Chen et al. [42] also reported a high-performance composite gel with a higher ratio of surface-deacetylated CHNF with a unique crosslinking reagent. They used porcine skin gelatin and calf bone gelatin. Ge et al. [43] used chitin whiskers to reinforce gelatin hydrogels derived from porcine skin. Their collagen sources are different from our study, and minor differences exist in the solvent used and the fiber ratios. In this study, we prepared photocrosslinked M-FCP hydrogels reinforced by CHNF (Scheme 1) for application as a biomaterial, specifically for wound-dressing materials and scaffolds for regenerative medicine. For medical applications, especially surgical operations, stand-alone hydrogel films of moderate toughness are useful for rapid handling during operations. Therefore, we investigated the mechanical properties of reinforced hydrogels as well as cell proliferation in these materials.

## 2. Materials and Methods

### 2.1. Materials

Commercial grade FCP (Ixos HDL-50SP; *M*_n_ ca. 5000 g/mol) was purchased from Nitta Gelatin Co., Ltd. (Tokyo, Japan). Chitin nanofibers (CHNFs) were prepared from crab shells by the nanofibrillation method, using a grinding machine, as described in a previous report [34]. Organic solvents for the synthesis and purification of M-FCP were used as received. For the synthesis of M-FCP, anhydride grade dimethylsulfoxide (DMSO) was used as purchased from Merck KGaA as the Sigma-Aldrich brand (St. Louis, MO, USA). Glycidyl methacrylate (GMA) and methanol were purchased from Kanto Chemical Co., Inc. (Tokyo, Japan). 2-Ethyl-4-methylimidazole (EMI) was purchased from Tokyo Chemical Industry, Co., Ltd. (Tokyo, Japan). Irgacure 2959 (2-hydroxy-1-[4-(hydroxyethoxy)phenyl]-2-methyl-1-propanone) was kindly provided by Ciba Specialty Chemicals K. K. (Now BASF Japan Ltd., Tokyo, Japan) and used as received. For the gel preparation, ultra-pure water (electrical resistance >18 MΩ cm^−1^) was obtained from a Millipore Direct-Q water purification system (Merck KGaA, Darmstadt, Germany). 3T3 Swiss albino fibroblasts were distributed at RIKEN Bioresource Research Center (Ibaraki, Japan). Dulbecco’s modified Eagle’s medium (DMEM), a mixed penicillin/streptomycin solution (of 10,000 units/mL penicillin and 10,000 μg/mL streptomycin), and 3-(4,5-dimethyl-2-thiazolyl)-2,5-diphenyltetrazolium bromide (MTT) were purchased from Fujifilm Wako Pure Chemical Co. (Osaka, Japan).

### 2.2. Synthesis of Methacrylate-Modified Fish Collagen Peptide (M-FCP)

M-FCP was synthesized according to our previously described method [29]. Twenty-five grams of FCP were dissolved in 300 mL of anhydrous DMSO. To this solution, 2.4 g of EMI was added, and then 84 mL of GMA was added dropwise. The solution was stirred at 28 ± 2 °C for 24 h. After the reaction, the solution was poured into 1.5 L of methanol, and the mixture was stirred for 2 h. The mixture was filtered through filter paper. The precipitate was washed with 1.5 L of methanol and then dissolved in 300 mL of water. After lyophilization from the aqueous solution, a white, foam-like solid of M-FCP was obtained (yield: 38%).

### 2.3. Preparation of M-FCP/CHNF Composite Hydrogel by Photoirradiation

The composite hydrogels of M-FCP with CHNF were prepared by photocrosslinking with a radical photoinitiator. First, 0.96% CHNF dispersion was added to water and sonicated for 30 s using a Branson Sonifier 250 ultrasonic homogenizer (Emerson Electric Co., St. Louis, MO, USA). M-FCP and Irgacure 2959 were dissolved in the CHNF dispersion. The final concentration of M-FCP in the solution was varied as 15 or 20 wt%, while that of CHNF was varied as 0.2, 0.4, or 0.6 wt% (Table 1). The hydrogel samples were named following this pattern: M-FCP15/CHNF0.2 for a composite hydrogel fabricated from an original solution containing 15 wt% M-FCP and 0.2 wt% CHNF. The final concentration of Irgacure 2959 was 0.05 wt%. To prepare sample specimens for the compression test, the solution was poured into a small Teflon sample vessel with an inner diameter of 10 mm and a height of 20 mm, then degassed for 30 min under reduced pressure before UV irradiation. To prepare the sample specimens for the tensile test, the solution was poured into a casting mold prepared from two stacked 100 mm × 100 mm silicone plates with a thickness of 1 mm, one of which had several holes with dimensions of 5 mm × 40 mm. The solution was degassed for 10 min under reduced pressure before UV irradiation. The solution was irradiated with UV light (~60 mW/cm^2^) for 450 s using a SpotCure SP-7 UV lamp unit (Ushio Inc., Tokyo, Japan) equipped with a homogeneous irradiation unit; the light was passed through a glass plate to eliminate light of wavelengths less than 300 nm. The gel samples for the compression test had a cylindrical shape with a diameter of 10 mm and a thickness of 10 mm, and those for the tensile test had a width of 5 mm, a length of 40 mm, and a thickness of 1 mm.

### 2.4. Characterization

The reactants and the product were analyzed by nuclear magnetic resonance (NMR) spectroscopy using an Avance III HD 400 MHz spectrometer (Bruker, Billerica, MA, USA). In the measurement of ^1^H NMR of FCP and M-FCP, the number of scans was 128, and deuterium oxide was used as a solvent, with sodium 3-(trimethylsilyl)propionate-2,2,3,3-*d*_4_ (TMSP) as an internal chemical-shift standard. In the measurement of ^1^H NMR of GMA, the number of scans was 16, and DMSO-*d*_6_ was used as a solvent, with tetramethylsilane (TMS) as an internal chemical-shift standard. For the general procedure, ca. 10 mg of each sample was dissolved in 600 μL of the corresponding solvent, and the solution was measured in a glass sample tube with a diameter of 5 mm.

The infrared (IR) spectra of the reactants and the products were recorded on a Shimadzu FT-IR 8400S spectrometer (Shimadzu Co., Kyoto, Japan), using the KBr-pellet method with a wavelength region of 400–4000 cm^−1^. The number of scans was 50. A solid sample was mixed with excess KBr and ground using an agate mortar and pestle. The sample pellet was pressed with a hand press machine. A liquid sample was dissolved in chloroform at a concentration of 1 mg/mL, and 5 μL of the solution was cast on the KBr pellet.

The mechanical properties of the hydrogel products were analyzed by a compression test and a tensile test. These tests were carried out using a Shimadzu EZ-S tabletop universal tester equipped with a 100-N load cell (Kyoto, Japan). The crosshead speed for the compression test was 1 mm/min, while that for the tensile test was 10 mm/min. Five specimens were used for each sample condition, and average and standard deviation values were calculated. Data are expressed as the average ± the standard deviation.

The morphology of the lyophilized gel was observed using a Hitachi S-4700 field emission scanning electron microscope (FE-SEM) (Tokyo, Japan). The cross-sectional surface of lyophilized gel was observed at the accelerating voltage of 2 kV after coating with gold using a Hitachi gold ion sputter.

### 2.5. Cell Proliferation Assay

The cell proliferation assay on the hydrogel thin films was carried out according to our previously described method [30]. The thin films of hydrogel were prepared on round-shaped glass coverslips (No. 1, Matsunami Glass Ind. Ltd., Osaka, Japan) with a diameter of 15 mm by the dip-coating method, followed by UV irradiation to crosslink M-FCP. The solution used in the dip-coating method was the same as used in the preparation of the corresponding hydrogel. After crosslinking, the coverslips were irradiated with UV light and placed into a 24-well polystyrene culture plate. The culture plate was sealed with a polyester sealing film and sterilized with 10 kGy γ-ray irradiation, which was conducted by Koga Isotope, Ltd. (Shiga, Japan). After washing the films on the coverslips with phosphate-buffered saline (PBS) and DMEM, 3T3 Swiss albino fibroblasts were seeded on the films at a concentration of 5 × 10^3^ cells/mL, with 300 μL of DMEM containing 1% penicillin/streptomycin and 10% fetal bovine serum (FBS) (J R Scientific, Inc., Woodland, CA, USA). Cells were incubated at 37 °C with 5% CO_2_ in a CO_2_ incubator (Astec Co., Ltd., Fukuoka, Japan). Cell proliferation was assayed by the conventional MTT method. In the procedure, 300 μL of 1.0 mg/mL MTT solution was added to the cells on each sample after growth and incubated at 37 °C for 90 min in the CO_2_ incubator. After the incubation, the sample was washed with 300 μL of PBS, and 300 μL of cell lysis solution (10% NP-40, polyoxyethylene (9) octylphenyl ether) was added to the sample. The absorbance of the solution at 570 nm was recorded on an iMark microplate reader (Bio-Rad, Hercules, CA, USA) after sufficient pipetting. The cell morphology was observed using a Carl-Zeiss Axio Vert. A1 phase-contrast microscope (Carl-Zeiss AG, Oberkochen, Germany). We used an image analysis method with ImageJ image processing software (open-source, developed at NIH) to check the circularity of cells. At least 30 cells after 3-day culture are analyzed for each sample.

### 2.6. Statistical Analysis

Mechanical property data and cell proliferation data are presented as mean ± standard deviation. Experiments to determine mechanical properties were repeated at least five times, and experiments to determine cell proliferation were repeated in triplicate. Statistical significance was determined using Welch’s *t*-distribution for assessing the difference between the two sample means. Because SD values for the compression strength were very high, we checked the normality with the Kolmogorov–Smirnov test and the Shapiro–Wilks test as a normality test using R software (an open-source language for statistical analysis supported by the R Foundation for Statistical Computing). As a result, it was found that all data retained normality.

## 3. Results and Discussion

### 3.1. Synthesis of Methacrylate-Modified Collagen Peptide (M-FCP)

FCP was easily dissolved in DMSO due to its relatively low molecular weight, and the reaction and subsequent purification were carried out smoothly (compared with gelatin). Figures S1 and S2 show the IR and NMR spectra of M-FCP, respectively (see the supplementary information). The synthesis of M-FCP was successful, and the NMR spectrum was consistent with our previous report [29]. The degree of modification of the amino groups was 51% when calculated from the NMR spectrum with the hypothesis that each amino group of all lysine residues can react with two molecules of GMA. On average, therefore, one methacrylate group was attached to each lysine residue of FCP. The product maintained good water solubility at room temperature and did not gel by simply cooling the solution.

### 3.2. Preparation of M-FCP/CHNF Composite Hydrogels by Photocrosslinking

**Figure 1:**
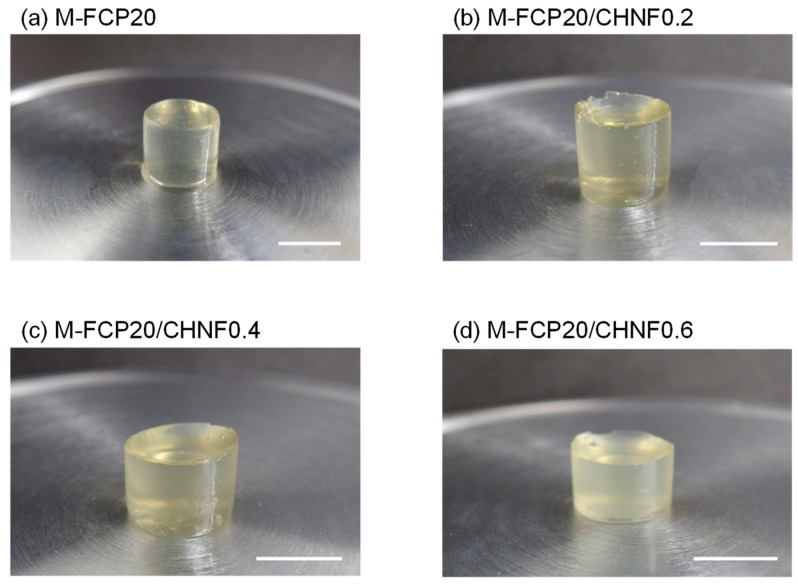
Digital photographs of composite hydrogels of M-FCP and CHNF: (**a**) M-FCP20, (**b**) M-FCP20/CHNF0.2, (**c**) M-FCP20/CHNF0.4, and (**d**) M-FCP20/CHNF0.6. The scale bar represents 10 mm.

**Figure 2:**
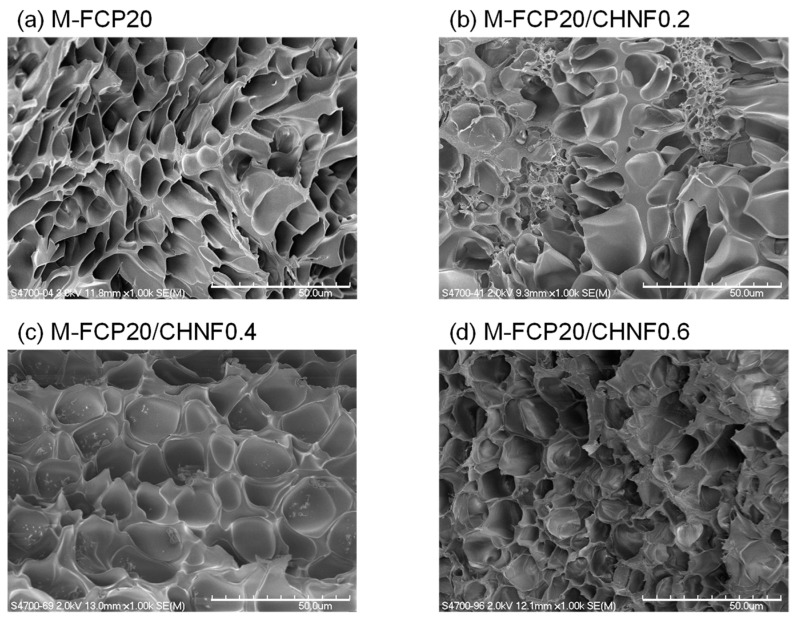
Morphologies of lyophilized composite gels observed with FE-SEM: (**a**) M-FCP20, (**b**) M-FCP20/CHNF0.2, (**c**) M-FCP20/CHNF0.4, and (**d**) M-FCP20/CHNF0.6. The scale bar represents 50 μm.

M-FCP was dissolved in water and photoirradiated in the presence of a radical photoinitiator. The solution containing CHNF was translucent before crosslinking, while the solution without CHNF was transparent. This was due to the existence of small bundles of CHNF and the difference in the refractive indexes of the M-FCP solution and CHNF. Photoirradiation for 450 s was sufficient to obtain solid gels for all samples with a CHNF concentration between 0 and 0.6 wt%, even when the UV light was irradiated only from the top of the solution with a height of 1 cm. Figure 1 shows photographs of the M-FCP20 hydrogels with and without CHNF. Although transparency was slightly decreased with an increase in CHNF concentration, the light was not blocked by this slight turbidity. Furthermore, this slight turbidity remained homogeneous and unchanged during photoirradiation, implying the fine dispersion of CHNF in the gel as well as in the solution. Though the turbidity depends on the content of CHNF in the gel, all of our hydrogels did not prevent the observation of cell morphologies using a conventional optical microscope. Figure 2 shows the morphologies of the lyophilized M-FCP20 hydrogels observed with FE-SEM. All lyophilized gels had a porous morphology, with pores of around 10 μm in size. The pore size of the lyophilized hydrogels was distributed from 3 to 20 μm, which did not change significantly with CHNF content. The pore size was originally the size of the ice crystals that grew during freezing in the first step of lyophilization. Based on our further consideration, the pore size of the ice crystals depended upon the concentration and heterogeneity of fillers. The interconnectivity of these pores was not observed. Rizwan et al. reported that the physical crosslinking of the photocrosslinkable gelatin gel by cooling at 4 °C showed smaller pores than without physical crosslinking [44]. Though rigid crosslinking can make the pore smaller, especially at the higher concentration of gelatin, such morphology change was not observed in our composite gel by the addition of a low concentration of CHNF. Though the reason has not been fully accounted for, the result implies that the interaction between CHNF and M-FCP was not so rigid but flexible, possibly due to the low concentration of CHNF. Therefore, CHNF did not affect the pore size. In our composite gels, the CHNF content did not affect the growth of ice crystals because of its very small size, low concentration, and relatively good dispersion. The fibrous morphology on the pore wall was not observed at this magnification.

### 3.3. Mechanical Properties of M-FCP/CHNF Composite Hydrogels

**Figure 3:**
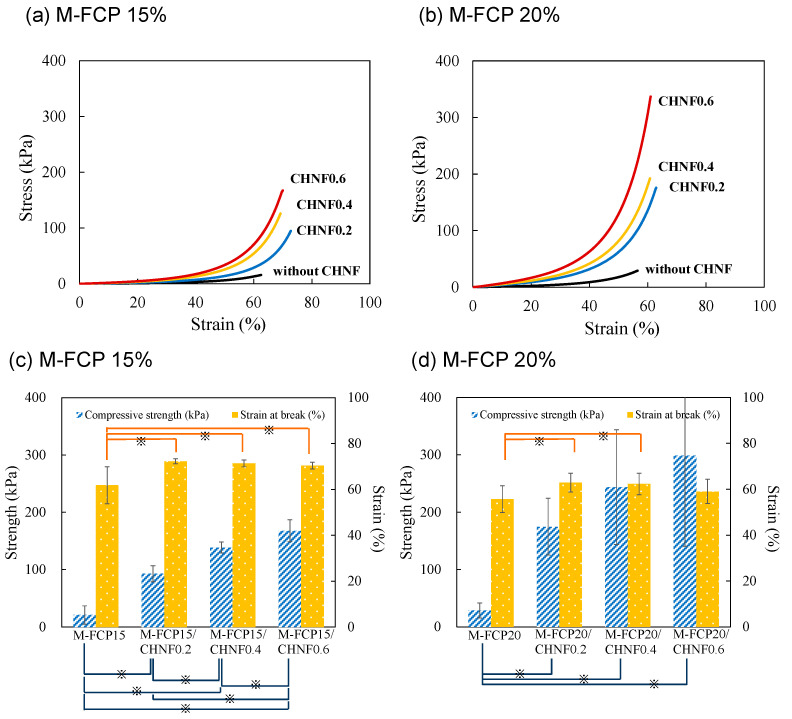
Stress–strain curves of M-FCP/CHNF composite hydrogels in the compression test ((**a**) M-FCP15 series and (**b**) M-FCP20 series). CHNF content was varied from 0 to 0.6%. The compression properties are summarized in the graphs ((**c**) M-FCP15 series and (**d**) M-FCP20 series). The columns represent mean values, and the error bars represent standard deviations (*n* = 5). The strength of the M-FCP/CHNF composite hydrogels was higher than that of the M-FCP hydrogels without CHNF at any CHNF content. Statistical significance was indicated by *p* < 0.05 (※).

The mechanical properties of the composite hydrogels were investigated using a compression test. The test was carried out at both concentrations of 15 and 20 wt% for M-FCP, while the concentration of CHNF was varied from 0 to 0.6 wt%. Figure 3a,b show the typical stress–strain curves for the compression test of the hydrogels with an M-FCP concentration of 15 and 20 wt%, respectively. Figure 3c,d summarize the mechanical properties from the compression test of the hydrogels with an M-FCP concentration of 15 and 20 wt%, respectively. The mechanical properties were significantly improved by the addition of CHNF (*p* < 0.05), and the gel strength, i.e., the stress at break, increased with an increase in CHNF content. Although the strain at break slightly increased with the addition of CHNF, the CHNF content did not influence the strain at break under conditions of CHNF content of more than 0.2 wt%. In a comparison of 15 wt% M-FCP hydrogels with 20 wt% M-FCP hydrogels, the strength of 20 wt% hydrogels was significantly higher than that of 15 wt% hydrogels at each content of CHNF (*p* < 0.05 for CHNF 0.2 and 0.4 wt%). However, the strain at break of 20 wt% hydrogels was lower than that of 15 wt% hydrogels (*p* < 0.05 for CHNF 0.2, 0.4, and 0.6 wt%). These results were attributed to the reinforcing effect of the CHNF and the intrinsic brittleness of hydrogel that was enhanced by the increase in the polymer density. A similar tendency of this brittleness was observed in our previous study of crosslinked fish gelatin [29]. The error bars show the standard deviations (SDs) of each condition. The error bars in Figure 3 indicated the existence of a very large SD in the compression strength of 20% M-FCP with a higher content of CHNF. We consider that these large SDs had two causes. First, the compression stress rapidly increased with the increase in strain, especially in the region of strain over 50%. Second, the hydrogels with higher CHNF content may have had intrinsic inhomogeneity, which could have been caused by an insufficient dispersion of CHNF.

**Figure 4:**
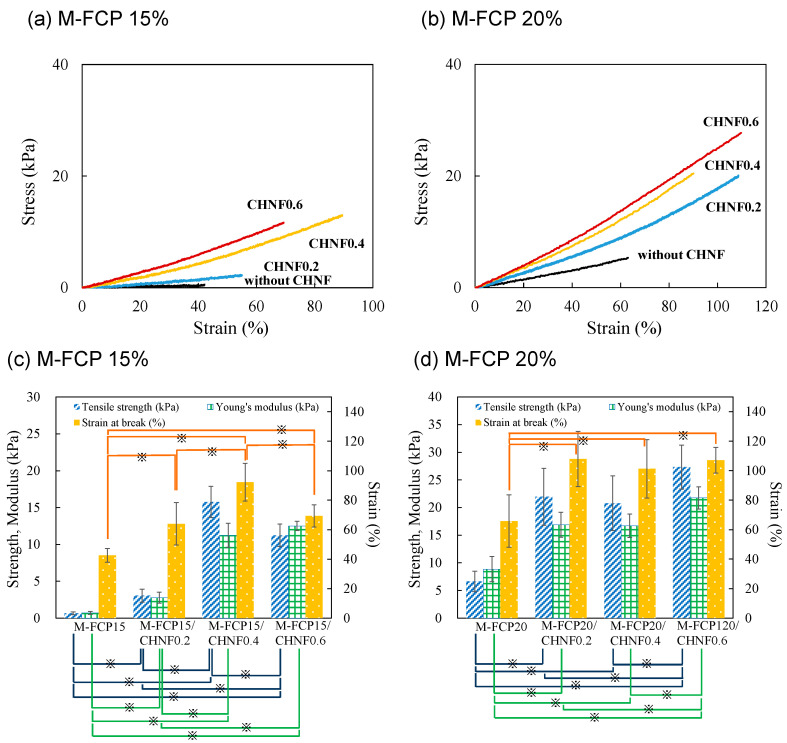
Stress–strain curves of M-FCP/CHNF composite hydrogels in the tensile test ((**a**) M-FCP15 series and (**b**) M-FCP20 series). The CHNF content was varied from 0 to 0.6%. The tensile properties are summarized in the graphs ((**c**) M-FCP15 series and (**d**) M-FCP20 series). The columns represent mean values, and the error bars represent standard deviations (*n* = 5). The strength of M-FCP/CHNF composite hydrogels was significantly higher than that of the M-FCP hydrogels without CHNF at any CHNF content. Statistical significance was indicated by *p* < 0.05 (※).

To verify the large error in the compression test of the hydrogels with higher CHNF content, we also investigated the tensile properties of the hydrogels. Figure 4a,b show the typical stress–strain curves for the tensile test of the hydrogels with 15 and 20 wt% M-FCP, respectively. Figure 4c,d summarize the mechanical properties for the tensile test of the hydrogels with 15 and 20 wt% M-FCP, respectively. The results revealed that the tensile properties of M-FCP hydrogels were also significantly reinforced by CHNF (*p* < 0.05). In contrast to the results of the compression tests, the strain at break (elongation) was increased by the presence of CHNF. This improvement of the elongation property is attributed to the reinforcement along the fiber axis of CHNF due to the interaction between M-FCP and CHNF. This improvement in the elongation property was also previously reported for microfibrillated cellulose-reinforced elastin peptide hydrogels [30]. From the results for M-FCP15, we observed a peak in the tensile strength at a CHNF content of 0.4%. The tensile strength and the strain at break of M-FCP15/CHNF0.6 were significantly lower than those of M-FCP15/CHNF0.4 (*p* < 0.05), although Young’s modulus of M-FCP15/CHNF0.6 was slightly higher than that of M-FCP15/CHNF0.4 (but not significantly). This result was considered to be due to the non-homogeneity of CHNF in the M-FCP15/CHNF0.6 samples. From the results for M-FCP20, we observed a plateau in the tensile strength at CHNF contents of 0.2 to 0.4%. This plateau implied the non-homogeneity of CHNF in the M-FCP20/CHNF0.4 samples. However, a significant improvement in these properties was observed in M-FCP20/CHNF0.6 (*p* < 0.05). We assumed that this improvement in M-FCP20/CHNF0.6 could be explained by the increase in the viscosity of the liquid at the mixing step, before crosslinking. It is considered that higher shear stress is applied to nanofibers in a more viscous liquid at the mixing step.

As previously described in the Introduction section, Hassanzadeh et al. [41] and Chen et al. [42] reported high-performance composite hydrogels with a higher ratio of CHNF. Although these two groups reported very high mechanical properties for their composite gels, we cannot compare our M-FCP hydrogels with their hydrogel composites because of the enormous difference in the CHNF contents and the molecular weight of the protein. Ge et al. [43] used chitin whiskers to reinforce gelatin hydrogels derived from porcine skin. Their hydrogels were weaker than our hydrogels in the compression test, perhaps due to the absence of chemical crosslinking.

### 3.4. Cell Proliferation on M-FCP/CHNF Composite Hydrogels

**Figure 5:**
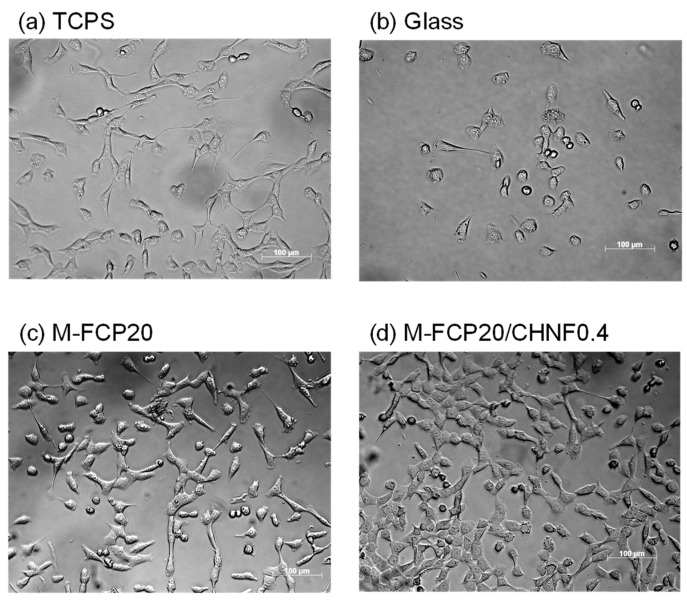
Microscopy images of fibroblasts grown on each sample for 5 days: (**a**) tissue culture polystyrene (TCPS), (**b**) glass coverslips washed with acid (Glass), (**c**) M-FCP20, and (**d**) M-FCP20/CHNF0.4.

**Figure 6:**
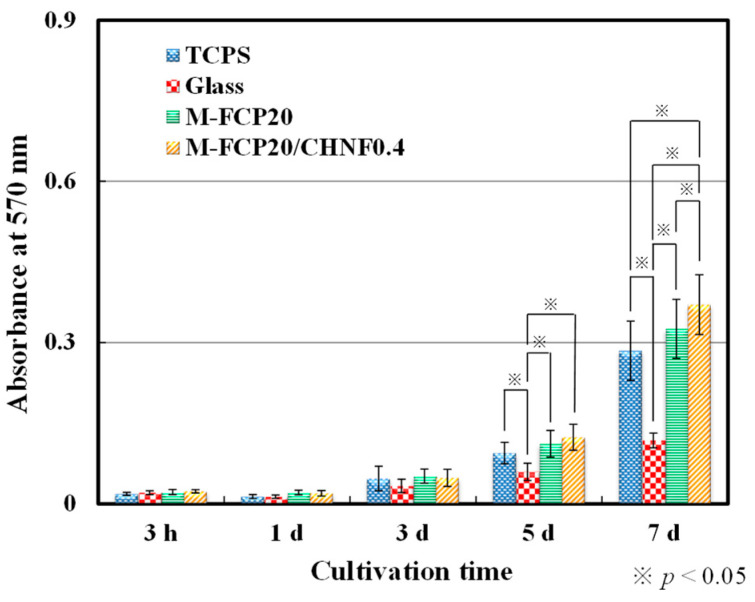
MTT assay of fibroblast growth on tissue culture polystyrene (TCPS), glass coverslips washed with acid (Glass), M-FCP20, and M-FCP20/CHNF0.4. The columns represent mean values, and the error bars represent standard deviations (*n* = 3). Statistical significance was indicated by *p* < 0.05 (※).

3T3 Swiss albino fibroblasts were seeded on glass coverslips covered with M-FCP hydrogel or M-FCP/CHNF hydrogel. Following incubation for a prescribed time, cell morphologies and growth were observed under a microscope. Figure 5 shows images of the results of the microscopy observations. Cells proliferated on both hydrogels, and the cell morphologies were the same as those of fibroblasts with good adhesion and proliferation reported previously [45]. We checked the circularity of cells and found that the average circularity of cells on M-FCP/CHMF (0.514 ± 0.202) was significantly lower than that of cells on glass (0.638 ± 0.163) (*p* < 0.05). The circularity of cells is one of the indicators of cell spreading via adhesion [46]. A lower value implies good adhesion. Figure 6 shows the results of the MTT assay, to quantitatively express the proliferation of cells [47]. According to the results of the MTT assay, we observed as much cell growth on M-FCP as on the tissue culture polystyrene. There was slightly more growth on the M-FCP/CHNF hydrogel in comparison with the growth on the M-FCP hydrogel (*p* < 0.05). This effect was due to the presence of CHNF, which is reported to promote the growth of vascular cells [41], keratinocytes [48], fibroblasts [48,49,50], myoblasts [51], and osteoblasts [51,52]. We consider the intrinsic reason for this effect may be attributed to the enhancement of the stiffness of the hydrogel. It is known that fibroblasts show good adhesion with extensive morphology on a harder surface [53,54,55]. As revealed by the results shown in Figure 4b, the increase in the modulus obviously follows the addition of CHNF. Furthermore, considering the small vicinal region around nanofibers from a microscopic viewpoint, the micro-regional modulus was thought to be higher than that of the bulk material that we measured in the tensile test, and cells could detect this micro-regional modulus because of their small size. Many researchers have noted the influence of nanofibers on the growth of fibroblasts [56,57,58]. According to our results, the composite materials produced only from marine resources can be applied as a biodegradable scaffold for use in regenerative medicine such as wound-dressing materials.

## 4. Conclusions

We prepared CHNF-reinforced FCP hydrogels by photocrosslinking at methacrylate groups chemically attached to FCP. We observed the homogeneous, porous morphology of the lyophilized hydrogels by FE-SEM. These observations revealed that there was no aggregation of CHNF at the micrometer level. The mechanical tests revealed that the compression and tensile properties of the FCP hydrogel were remarkably improved. The tensile strength and modulus increased with an increase in CHNF content. In the cell culture assay, fibroblasts proliferated well on the CHNF-reinforced FCP hydrogel, which implies that the hydrogel we developed in our study, using only marine resources, is a good candidate for scaffold material for fibroblasts in connective tissues.

## Figures and Tables

**Table 1:** Quantities of components for the preparation of M-FCP/CHNF composite hydrogels. The percentage contents are provided in parentheses.

Sample	Water	M-FCP	0.96% CHNF Dispersion	Irgacure 2959
M-FCP15	8.45 g	1.50 g (15%)	-	0.05 g (0.5%)
M-FCP15/CHNF0.2	6.37 g	1.50 g (15%)	2.08 g (0.2%)	0.05 g (0.5%)
M-FCP15/CHNF0.4	4.28 g	1.50 g (15%)	4.17 g (0.4%)	0.05 g (0.5%)
M-FCP15/CHNF0.6	2.20 g	1.50 g (15%)	6.25 g (0.6%)	0.05 g (0.5%)
M-FCP20	7.95 g	2.00 g (20%)	-	0.05 g (0.5%)
M-FCP20/CHNF0.2	5.87 g	2.00 g (20%)	2.08 g (0.2%)	0.05 g (0.5%)
M-FCP20/CHNF0.4	3.75 g	2.00 g (20%)	4.17 g (0.4%)	0.05 g (0.5%)
M-FCP20/CHNF0.6	1.70 g	2.00 g (20%)	6.25 g (0.6%)	0.05 g (0.5%)

## Data Availability

The data presented in this study are available on request from the corresponding author.

## Supplementary Materials

The following are available online at https://www.mdpi.com/article/10.3390/polym15030682/s1, Figure S1: IR spectrum of M-FCP, Figure S2: NMR spectrum of M-FCP, Figure S3: NMR spectrum of M-FCP in D2O, Figure S4: NMR spectrum of FCP in D2O, Figure S5: NMR spectrum of GMA in DMSO-d6, Figure S6: Digital photographs of composite hydrogels of M-FCP and CHNF: (a) M-FCP20, (b) M-FCP20/CHNF0.2, (c) M-FCP20/CHNF0.4, and (d) M-FCP20/CHNF0.6 for the tensile test. The scale bar represents 10 mm and Table S1: Circularity of cells on each sample calculated using an ImageJ software.

## References

[1] Anderson J.L., Asche F., Garlock T. (2018). Globalization and commoditization: The transformation of the seafood market. J. Commod. Mark..

[2] Kim S.K., Mendis E. (2006). Bioactive compounds from marine processing byproducts-A review. Food Res. Int..

[3] Kim S.K., Kim Y.T., Byun H.G., Nam K.S., Joo D.S., Shahidi F. (2001). Isolation and characterization of antioxidative peptides from gelatin hydrolysate of Alaska pollack skin. J. Agric. Food Chem..

[4] Byun H.G., Kim S.K. (2001). Purification and characterization of angiotensin I converting enzyme (ACE) inhibitory peptides from Alaska pollack (*Theragra chalcogramma*) skin. Process Biochem..

[5] Hong H., Fan H., Chalamaiah M., Wu J. (2019). Preparation of low-molecular-weight, collagen hydrolysates (peptides): Current progress, challenges, and future perspectives. Food Chem..

[6] Morimura S., Nagata H., Uemura Y., Fahmi A., Shigematsu T., Kida K. (2002). Development of an effective process for utilization of collagen from livestock and fish waste. Process Biochem..

[7] Saito M., Kiyose C., Higuchi T., Uchida N., Suzuki H. (2009). Effect of collagen hydrolysates from salmon and trout skins on the lipid profile in rats. J. Agric. Food Chem..

[8] Kobayashi K., Maehata Y., Kawamura Y., Kusubata M., Hattori S., Tanaka K., Miyamoto C., Yoshino F., Yoshida A., Wada-Takahashi S. (2011). Direct assessments of the antioxidant effects of the novel collagen peptide on reactive oxygen species using electron spin resonance spectroscopy. J. Pharmacol. Sci..

[9] Wang Y., Zhang C., Zhang Q., Li P. (2011). Composite electrospun nanomembranes of fish scale collagen peptides/chito-oligosaccharides: Antibacterial properties and potential for wound dressing. Int. J. Nanomed..

[10] Zhang C., Yang X., Hu W., Han X., Fan L., Tao S. (2020). Preparation and characterization of carboxymethyl chitosan/collagen peptide/oxidized konjac composite hydrogel. Int. J. Biol. Macromol..

[11] Lu S., Kong S., Wang Y., Hu Z., Zhang L., Liao M. (2022). Gastric acid-response chitosan/alginate/tilapia collagen peptide composite hydrogel: Protection effects on alcohol-induced gastric mucosal injury. Carbohydr. Polym..

[12] Ouyang Q.-Q., Hu Z., Lin Z.-P., Quan W.-Y., Deng Y.-F., Li S.-D., Li P.-W., Chen Y. (2018). Chitosan hydrogel in combination with marine peptides from tilapia for burns healing. Int. J. Biol. Macromol..

[13] Ohnishi A., Osaki T., Matahira Y., Tsuka T., Imagawa T., Okamoto Y., Minami S. (2013). Correlation of Plasma Amino Acid Concentrations and Chondroprotective Effects of Glucosamine and Fish Collagen Peptide on the Development of Osteoarthritis. J. Vet.-Med. Sci..

[14] Liu C., Sun J. (2015). Hydrolyzed tilapia fish collagen induces osteogenic differentiation of human periodontal ligament cells. Biomed. Mater..

[15] Raabe O., Reich C., Wenisch S., Hild A., Burg-Roderfeld M., Siebert H.-C., Arnhold S. (2010). Hydrolyzed fish collagen induced chondrogenic differentiation of equine adipose tissue-derived stromal cells. Histochem. Cell Biol..

[16] Li H., Wang D.Q., Chen H.L., Liu B.L., Gao L.Z. (2003). A Novel Gelatin-carbon Nanotubes Hybrid Hydrogel. Macromol. Biosci..

[17] Gelli R., Del Buffa S., Tempesti P., Bonini M., Ridi F., Baglioni P. (2018). Enhanced formation of hydroxyapatites in gelatin/imogolite macroporous hydrogels. J. Colloid Interface Sci..

[18] Sadat-Shojai M., Khorasani M.-T., Jamshidi A. (2015). 3-Dimensional cell-laden nano-hydroxyapatite/protein hydrogels for bone regeneration applications. Mater. Sci. Eng. C.

[19] Lee W.-F., Lee S.-C. (2006). Effect of hydrotalcite on the swelling and mechanical behaviors for the hybrid nanocomposite hydrogels based on gelatin and hydrotalcite. J. Appl. Polym. Sci..

[20] Hiraoka Y., Kimura Y., Ueda H., Tabata Y. (2003). Fabrication and Biocompatibility of Collagen Sponge Reinforced with Poly(glycolic acid) Fiber. Tissue Eng..

[21] Dash R., Foston M., Ragauskas A.J. (2013). Improving the mechanical and thermal properties of gelatin hydrogels cross-linked by cellulose nanowhiskers. Carbohydr. Polym..

[22] Wang W., Zhang X., Teng A., Liu A. (2017). Mechanical reinforcement of gelatin hydrogel with nanofiber cellulose as a function of percolation concentration. Int. J. Biol. Macromol..

[23] Lee D.H., Tamura A., Arisaka Y., Seo J.H., Yui N. (2019). Mechanically reinforced gelatin hydrogels by introducing slidable supramolecular cross-linkers. Polymers.

[24] Nakayama A., Kakugo A., Gong J.P., Osada Y., Takai M., Erata T., Kawano S. (2004). High Mechanical Strength Double-Network Hydrogel with Bacterial Cellulose. Adv. Funct. Mater..

[25] Luo K., Yang Y., Shao Z. (2016). Physically Crosslinked Biocompatible Silk-Fibroin-Based Hydrogels with High Mechanical Performance. Adv. Funct. Mater..

[26] Zhang L., Liu J., Zheng X., Zhang A., Zhang X., Tang K. (2019). Pullulan dialdehyde crosslinked gelatin hydrogels with high strength for biomedical applications. Carbohydr. Polym..

[27] Nojima T., Iyoda T. (2018). Egg white-based strong hydrogel via ordered protein condensation. NPG Asia Mater..

[28] Thakur S., Govender P.P., Mamo M.A., Tamulevicius S., Thakur V.K. (2017). Recent progress in gelatin hydrogel nanocomposites for water purification and beyond. Vacuum.

[29] Teramoto N., Hayashi A., Yamanaka K., Sakiyama A., Nakano A., Shibata M. (2012). Preparation and Mechanical Properties of Photo-Crosslinked Fish Gelatin/Imogolite Nanofiber Composite Hydrogel. Materials.

[30] Yano S., Mori M., Teramoto N., Iisaka M., Suzuki N., Noto M., Kaimoto Y., Kakimoto M., Yamada M., Shiratsuchi E. (2015). Preparation of Photocrosslinked Fish Elastin Polypeptide/Microfibrillated Cellulose Composite Gels with Elastic Properties for Biomaterial Applications. Mar. Drugs.

[31] Min B.M., Lee S.W., Lim J.N., You Y., Lee T.S., Kang P.H., Park W.H. (2004). Chitin and chitosan nanofibers: Electrospinning of chitin and deacetylation of chitin nanofibers. Polymer (Guildf).

[32] Zhao H.-P., Feng X.-Q., Gao H. (2007). Ultrasonic technique for extracting nanofibers from nature materials. Appl. Phys. Lett..

[33] Ifuku S., Nogi M., Abe K., Yoshioka M., Morimoto M., Saimoto H., Yano H. (2009). Preparation of chitin nanofibers with a uniform width as α-chitin from crab shells. Biomacromolecules.

[34] Salaberria A.M., Fernandes S.C.M., Diaz R.H., Labidi J. (2015). Processing of α-chitin nanofibers by dynamic high pressure homogenization: Characterization and antifungal activity against *A. niger*. Carbohydr. Polym..

[35] Zhong C., Cooper A., Kapetanovic A., Fang Z., Zhang M., Rolandi M. (2010). A facile bottom-up route to self-assembled biogenic chitin nanofibers. Soft Matter.

[36] Marchessault R.H., Morehead F.F., Walter N.M. (1959). Liquid Crystal Systems from Fibrillar Polysaccharides. Nature.

[37] Fan Y., Saito T., Isogai A. (2008). Chitin nanocrystals prepared by TEMPO-mediated oxidation of α-chitin. Biomacromolecules.

[38] Kadokawa J., Takegawa A., Mine S., Prasad K. (2011). Preparation of chitin nanowhiskers using an ionic liquid and their composite materials with poly(vinyl alcohol). Carbohydr. Polym..

[39] Ifuku S., Saimoto H. (2012). Chitin nanofibers: Preparations, modifications, and applications. Nanoscale.

[40] Ding F., Deng H., Du Y., Shi X., Wang Q. (2014). Emerging chitin and chitosan nanofibrous materials for biomedical applications. Nanoscale.

[41] Hassanzadeh P., Kazemzadeh-Narbat M., Rosenzweig R., Zhang X., Khademhosseini A., Annabi N., Rolandi M. (2016). Ultrastrong and flexible hybrid hydrogels based on solution self-assembly of chitin nanofibers in gelatin methacryloyl (GelMA). J. Mater. Chem. B.

[42] Chen C., Li D., Yano H., Abe K. (2019). Insect Cuticle-Mimetic Hydrogels with High Mechanical Properties Achieved via the Combination of Chitin Nanofiber and Gelatin. J. Agric. Food. Chem..

[43] Ge S., Liu Q., Li M., Liu J., Lu H., Li F., Zhang S., Sun Q., Xiong L. (2018). Enhanced mechanical properties and gelling ability of gelatin hydrogels reinforced with chitin whiskers. Food Hydrocoll..

[44] Rizwan M., Peh G.S.L., Ang H.P., Lwin N., Adnan K., Mehta J., Tan W., Yim E. (2017). Sequentially-crosslinked bioactive hydrogels as nano-patterned substrates with customizable stiffness and degradation for corneal tissue engineering applications. Biomaterials.

[45] Van Kooten T.G., Spijker H.T., Busscher H.J. (2004). Plasma-treated polystyrene surfaces: Model surfaces for studying cell-biomaterial interactions. Biomaterials.

[46] Wang Z., Guo Y., Zhang P. (2021). A rapid quantitation of cell attachment and spreading based on digital image analysis: Application for cell affinity and compatibility assessment of synthetic polymers. Mater. Sci. Eng. C.

[47] Zund G., Ye Q., Hoerstrup S.P., Schoeberlein A., Schmid A.C., Grunenfelder J., Vogt P., Turina M. (1999). Tissue engineering in cardiovascular surgery: MTT, a rapid and reliable quantitative method to assess the optimal human cell seeding on polymeric meshes. Eur. J. Cardio-Thorac. Surg..

[48] Noh H.K., Lee S.W., Kim J.M., Oh J.E., Kim K.H., Chung C.P., Choi S.C., Park W.H., Min B.M. (2006). Electrospinning of chitin nanofibers: Degradation behavior and cellular response to normal human keratinocytes and fibroblasts. Biomaterials.

[49] Ogawa Y., Azuma K., Izawa H., Morimoto M., Ochi K., Osaki T., Ito N., Okamoto Y., Saimoto H., Ifuku S. (2017). Preparation and biocompatibility of a chitin nanofiber/gelatin composite film. Int. J. Biol. Macromol..

[50] Noda T., Hatakeyama M., Kitaoka T. (2022). Combination of Polysaccharide Nanofibers Derived from Cellulose and Chitin Promotes the Adhesion, Migration and Proliferation of Mouse Fibroblast Cells. Nanomaterials.

[51] Wang L., Ezazi N.Z., Liu L., Ajdary R., Xiang W., Borghei M., Santos H.A., Rojas O.J. (2020). Microfibers synthesized by wet-spinning of chitin nanomaterials: Mechanical, structural and cell proliferation properties. RSC Adv..

[52] Tao F., Cheng Y., Shi X., Zheng H., Du Y., Xiang W., Deng H. (2020). Applications of chitin and chitosan nanofibers in bone regenerative engineering. Carbohydr. Polym..

[53] Choquet D., Felsenfeld D.P., Sheetz M.P. (1997). Extracellular matrix rigidity causes strengthening of integrin-cytoskeleton linkages. Cell.

[54] Yeung T., Georges P.C., Flanagan L.A., Marg B., Ortiz M., Funaki K., Zahir N., Ming W., Weaver V., Janmey P.A. (2005). Effects of substrate stiffness on cell morphology, cytoskeletal structure, and adhesion. Cell Motil. Cytoskelet..

[55] Hadjipanayi E., Mudera V., Brown R.A. (2009). Close dependence of fibroblast proliferation on collagen scaffold matrix stiffness. J. Tissue Eng. Regen. Med..

[56] Min B.M., Lee G., Kim S.H., Nam Y.S., Lee T.S., Park W.H. (2004). Electrospinning of silk fibroin nanofibers and its effect on the adhesion and spreading of normal human keratinocytes and fibroblasts in vitro. Biomaterials.

[57] Tian F., Hosseinkhani H., Hosseinkhani M., Khademhosseini A., Yokoyama Y., Estrada G.G., Kobayashi H. (2008). Quantitative analysis of cell adhesion on aligned micro- and nanofibers. J. Biomed. Mater. Res. Part A.

[58] Chen R.-D., Huang C.-F., Hsu S. (2019). Composites of waterborne polyurethane and cellulose nanofibers for 3D printing and bioapplications. Carbohydr. Polym..

