# Supplementary material for: Photocrosslinked Fish Collagen Peptide/Chitin Nanofiber Composite Hydrogels from Marine Resources: Preparation, Mechanical Properties, and an In Vitro Study
Source: Polymers (Basel). 2023 Jan 29;15(3):682. doi: 10.3390/polym15030682 (PMC9920125; doi:10.3390/polym15030682)
Supplement: Supplementary file 1 [file polymers-15-00682-s001.zip › polymers-2011371-supplementary.pdf]

Supplementary information for:

Photocrosslinked Fish Collagen Peptide/Chitin Nanofiber Composite Hydrogels from Marine Resources: Preparation, Mechanical Properties, and an *in vitro* study

Shinya Yano,[1] Kei Yamaguchi,[1] Mitsuhiro Shibata[1], Shinsuke Ifuku,[2] and Naozumi Teramoto[1,*]

[1]*Department of Applied Chemistry, Faculty of Engineering, Chiba Institute of Technology, 2-17-1 Tsudanuma, Narashino, Chiba 275-0016, Japan*

[2]*Department of Chemistry and Biotechnology, Graduate School of Engineering, Tottori University, 4-101 Koyama-cho Minami, Tottori, Tottori 680-8550, Japan*

* Corresponding author. Tel.: +81-47-478-0406; Fax: +81-47-478-0406

E-mail address: teramoto.naozumi@it-chiba.ac.jp

Department of Applied Chemistry, Faculty of Engineering, Chiba Institute of Technology, 2-17-1 Tsudanuma, Narashino, Chiba 275-0016, Japan

Figure S1: IR spectrum of M-FCP.

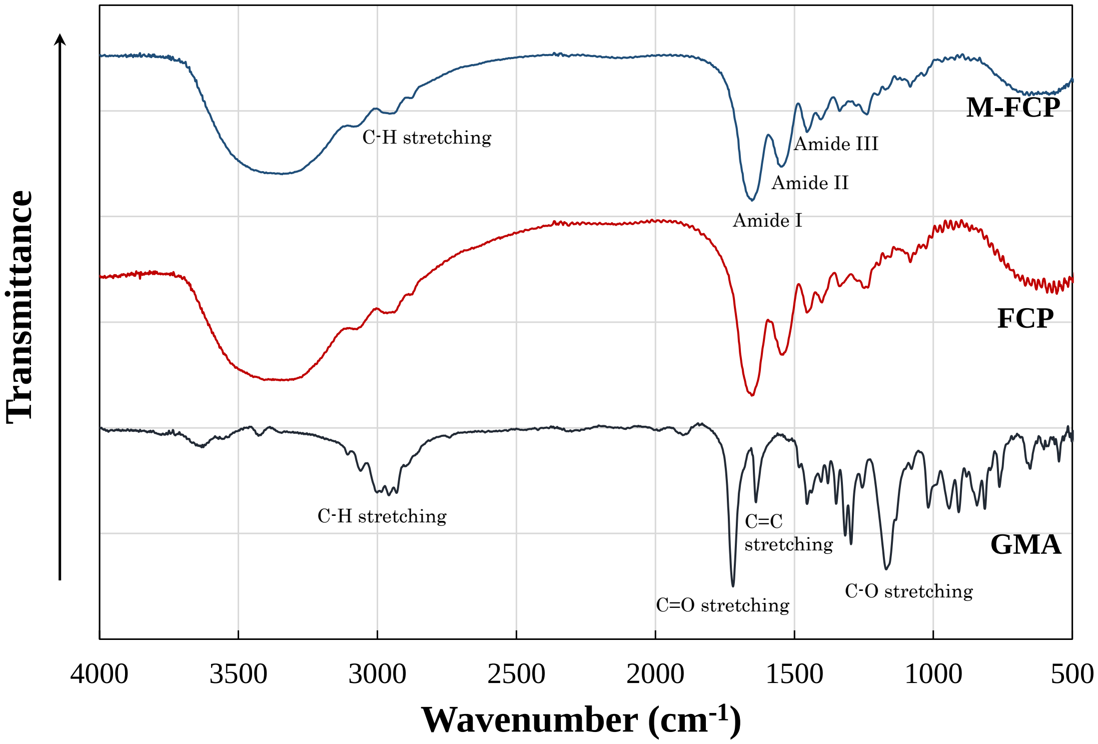

The absorption corresponding to methacrylate groups was not detected (less than noise level), because the amount of methacrylate groups attached to FCP was very low compared to the amount of FCP.

We tried to detect methacrylate groups by the calculation of differential spectrum between M-FCP and FCP. Figure S2 shows the differential spectrum. Though there is a lot of noise, we can observe the suspected peaks corresponding to C=O and C=C stretching vibration of methacrylate groups around 1750 cm^{-1} and 1650 cm^{-1}, respectively.

Figure S2: IR differential spectrum of M-FCP and FCP.

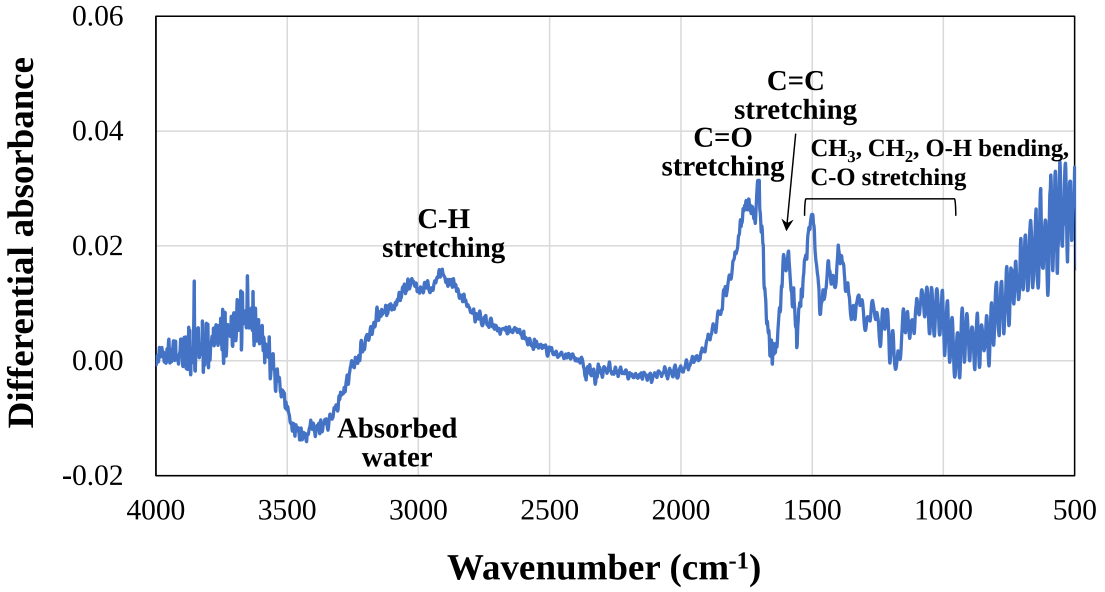

Figure S3: NMR spectrum of M-FCP in D_2O

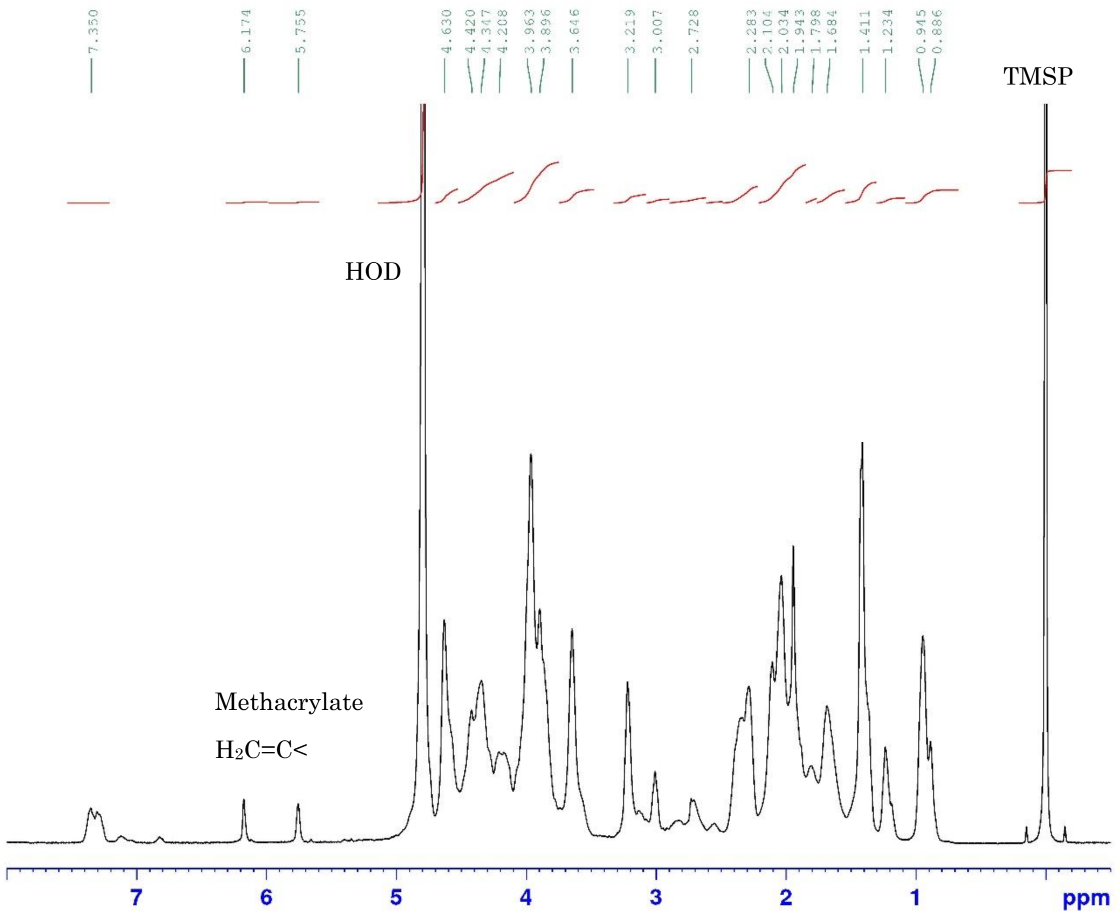

Signals corresponding to methacrylate were observed in the spectrum. The integral values of these signals were used for calculation of the degree of the modification. Sodium 3-(trimethylsilyl)propionate-2,2,3,3-d_4 (TMSP) was used as an internal chemical-shift standard.

Figure S4: NMR spectrum of FCP in D_2O

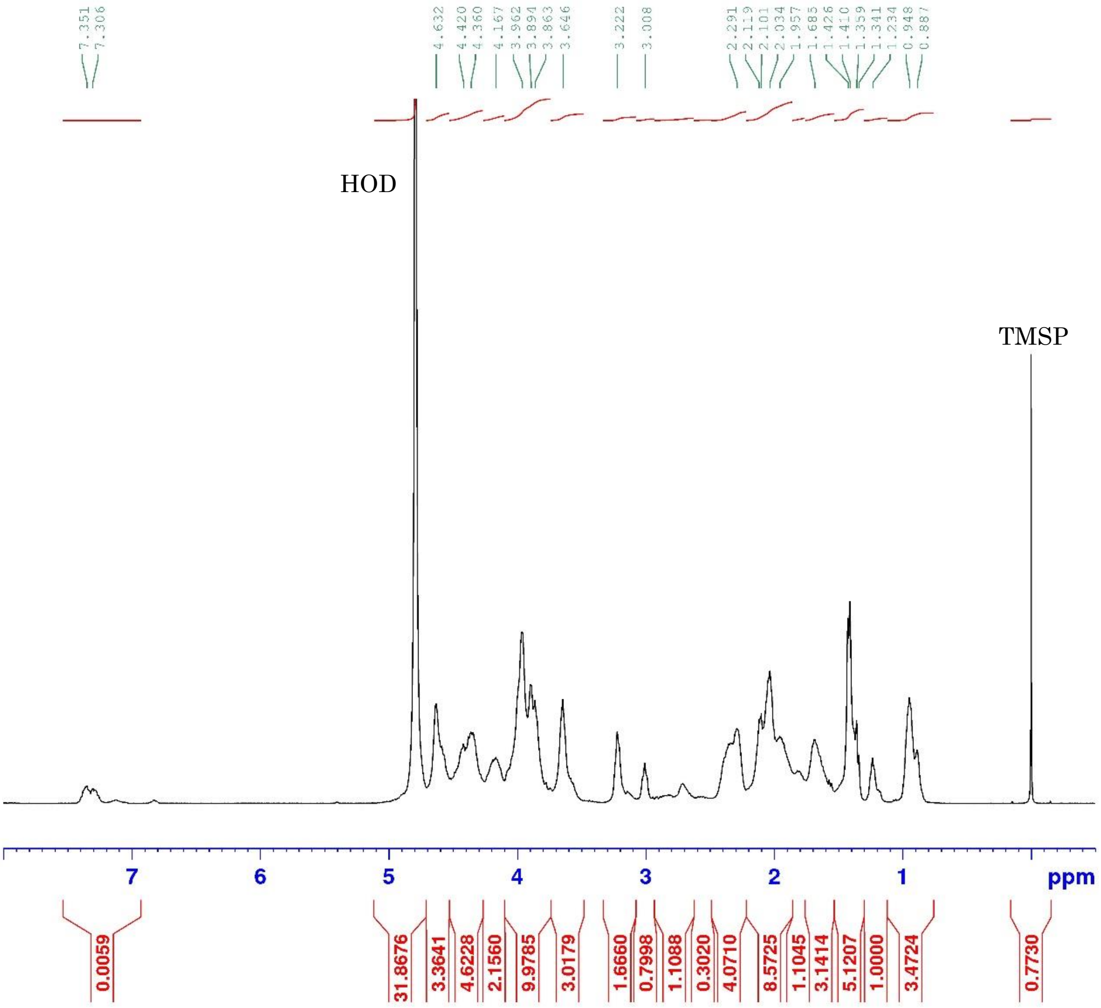

Referential spectrum for M-FCP synthesis.

Figure S5: NMR spectrum of GMA in DMSO-d_6

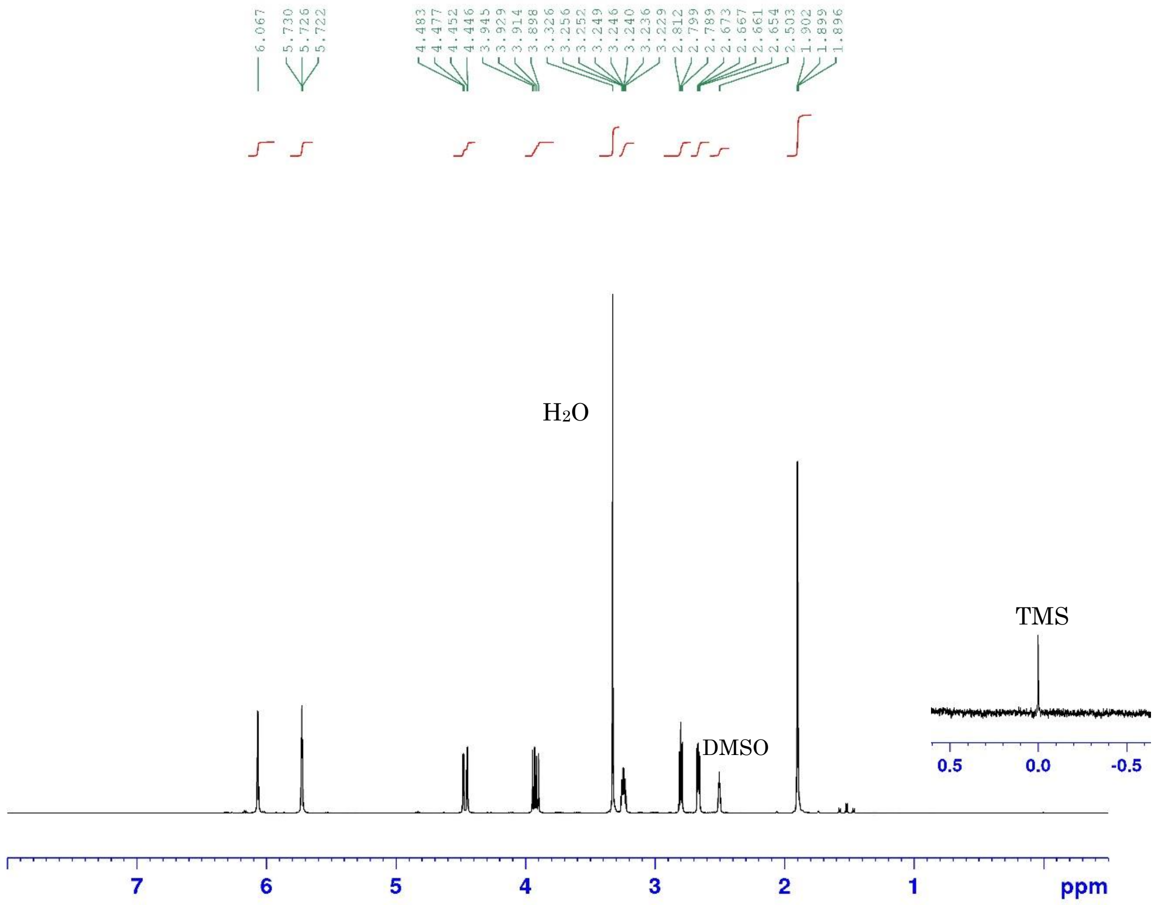

Referential spectrum for M-FCP synthesis.

Figure S6: Digital photographs of composite hydrogels of M-FCP and CHNF: (a) M-FCP20, (b) M-FCP20/CHNF0.2, (c) M-FCP20/CHNF0.4, and (d) M-FCP20/CHNF0.6 for the tensile test. The scale bar represents 10 mm.

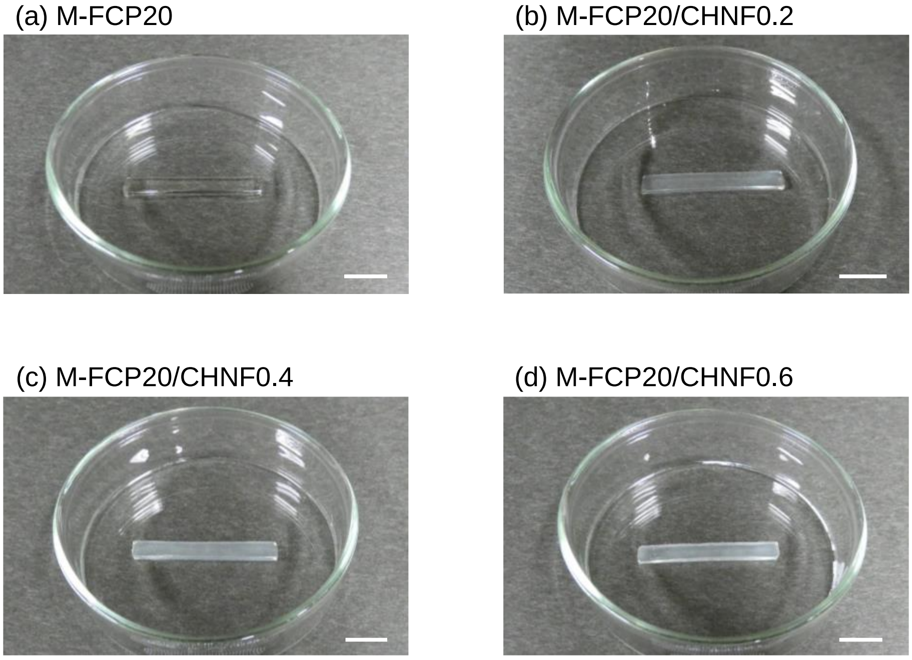

Table S1: Circularity of cells on each sample calculated using an ImageJ software.

	TCPS	Glass	M-FCP20	M-FCP20/CHNF0.4
Circularity	0.569 ± 0.194	0.638 ± 0.163	0.579 ± 0.234	0.514 ± 0.202

Ref: Wang Z, Guo Y, Zhang P. A rapid quantitation of cell attachment and spreading based on digital image analysis: Application for cell affinity and compatibility assessment of synthetic polymers. *Mater. Sci. Eng. C Mater. Biol. Appl.* **2021**, 128, 112267. doi:10.1016/j.msec.2021.112267
